# Genetic basis of the historical iron‐accumulating *dgl* and *brz* mutants in pea

**DOI:** 10.1111/tpj.16514

**Published:** 2023-10-26

**Authors:** Sophie A. Harrington, Marina Franceschetti, Janneke Balk

**Affiliations:** ^1^ Department of Biochemistry and Metabolism John Innes Centre Norwich NR4 7UH UK; ^2^ School of Biological Sciences University of East Anglia Norwich NR4 7TJ UK

**Keywords:** iron, hemerythrin, RNA‐seq, *Pisum sativum*, *Medicago truncatula*

## Abstract

The *Pisum sativum* (pea) mutants *degenerate leaves* (*dgl*) and *bronze* (*brz*) accumulate large amounts of iron in leaves. First described several decades ago, the two mutants have provided important insights into iron homeostasis in plants but the underlying mutations have remained unknown. Using exome sequencing we identified an in‐frame deletion associated with *dgl* in a *BRUTUS* homolog. The deletion is absent from wild type and the original parent line. BRUTUS belongs to a small family of E3 ubiquitin ligases acting as negative regulators of iron uptake in plants. The *brz* mutation was previously mapped to chromosome 4, and superimposing this region to the pea genome sequence uncovered a mutation in *OPT3*, encoding an oligopeptide transporter with a plant‐specific role in metal transport. The causal nature of the mutations was confirmed by additional genetic analyses. Identification of the mutated genes rationalizes many of the previously described phenotypes and provides new insights into shoot‐to‐root signaling of iron deficiency. Furthermore, the non‐lethal mutations in these essential genes suggest new strategies for biofortification of crops with iron.

## Significance Statement

Two iron‐accumulating pea mutants first described more than 30 years ago have greatly contributed to our understanding of iron homeostasis in plants, but the mutations were never identified. Here we show that the phenotypes are caused by mutations in the *BRUTUS* and *OPT3* genes and discuss how this leads to specific defects in iron signaling and leaf development.

## INTRODUCTION

**Figure 1:**
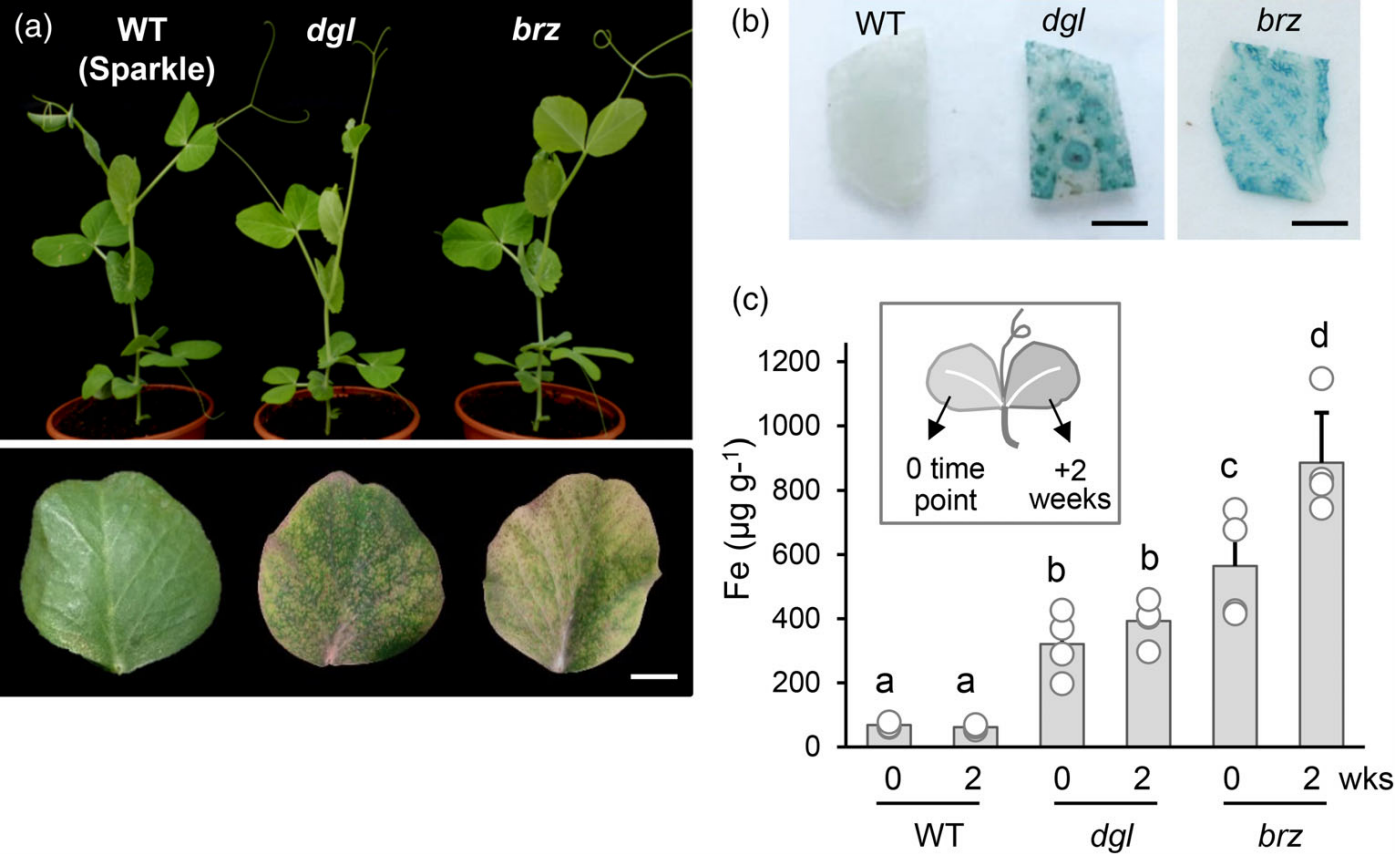
The *dgl* and *brz* mutants in pea (*Pisum sativum* L.) hyper‐accumulate iron in leaves. (a) Top panel: Three‐week old plants of the pea variety Sparkle used as wild type (WT) and the mutants *dgl* and *brz* in the Sparkle genetic background. Bottom panel: A lower leaflet of 5‐week‐old plants, showing yellowing and brown spots in the mutants. Scale bar = 1 cm. (b) Iron staining (blue) of leaf pieces from 5‐week‐old plants. Scale bar = 2 mm. (c) Iron concentration (μg per g dry weight) in leaflets of the second fully developed leaf, measured 2 weeks apart, using a colorimetric assay. Bars represent the mean of *n* = 3 (WT) or *n* = 4 (*dgl*, *brz*) values, which are shown as dots. Error bars represent SD. Letters indicate statistical differences using Student's *t*‐test of the mean.

Prior to the rise of Arabidopsis as a plant model organism, genetic studies in pea contributed greatly to our understanding of the mechanisms of inheritance and also helped identify several genetic loci for morphological and nutritional traits in plants (Ellis et al., 2011). As part of these studies, two iron‐accumulating pea mutants were independently isolated: *dgl* (*degenerate leaves*), from an X‐ray mutagenized population (Gottschalk, 1987), and *brz* (*bronze*), also named E107, generated using ethyl methanesulfonate (EMS) (Kneen et al., 1990). In addition to a root nodulation phenotype, the leaves of both mutants develop striking bronze spots and senesce prematurely (Figure 1a, bottom panel). The spots are necrotic tissue caused by toxic levels of iron, which accumulates 10 to 100‐fold in older leaves (Welch & LaRue, 1990; Figure 1b,c). Despite the similar phenotypes, genetic analysis showed that the *brz* and *dgl* loci segregate independently (Kneen et al., 1990). The *brz* mutation was mapped to a large segment of chromosome 4 but was not further fine‐mapped or cloned. For *dgl*, difficulties with distinguishing heterozygotes within the F2 population due to variability in iron concentration obstructed efforts to get an idea of its genome location (Kneen et al., 1990).

Despite not knowing the mutated genes, seminal physiological studies of the *dgl* and *brz* mutants demonstrated the existence of a Fe^3+^ reductase activity induced by iron deficiency (Grusak et al., 1990; Welch & LaRue, 1990) well before the corresponding gene was cloned from Arabidopsis (Robinson et al., 1999). In addition, grafting wild‐type shoots onto mutant roots and vice versa revealed the existence of a shoot‐to‐root signal for iron deficiency (García et al., 2013; Grusak & Pezeshgi, 1996; Welch & LaRue, 1990), the molecular nature of which is still unknown.

Because iron is toxic and controlled by tight homeostasis mechanisms, there is limited genetic variation for the iron concentration in crops and mutant screens rarely turn up iron‐accumulating mutants (Connorton & Balk, 2019; Lahner et al., 2003). Therefore, identifying the *dgl* and *brz* mutations could be important both for our general understanding of iron homeostasis and to help design strategies for biofortifying crops.

Finding genetic loci is greatly facilitated by a genome sequence, but owing to its large size (~4.45 Gb), a draft of the pea genome was not published until recently (Kreplak et al., 2019). This confirmed the close relationship and extensive co‐localization of genetic loci (synteny) with the *Medicago truncatula* genome. Making use of this new resource, we show that *dgl* is caused by a short deletion in *BRUTUS*, a putative iron sensor and negative regulator of iron uptake and that the *brz* phenotype is associated with mutations in the iron transporter *OPT3* in both pea and Medicago.

## RESULTS AND DISCUSSION

### *dgl* is associated with a small in‐frame deletion in *BRUTUS*

To investigate the transcriptional basis of iron accumulation in the *dgl* pea mutant and, if possible, to identify the causative mutation, we carried out RNA sequencing on leaves of *dgl* and the wild‐type cultivar Sparkle, into which the original *dgl* mutation was introgressed by at least five backcrosses (Marentes & Grusak, 1998). We found 86 differentially expressed genes which were highly enriched for genes involved in iron homeostasis (Table S1 and S2). These included four different ferritin genes with massively induced expression, in agreement with previous reports of increased ferritin protein in *dgl* leaves and seeds by electron microscopy, Western blot analysis, and iron‐stained native gels (Becker et al., 1998; Marentes & Grusak, 1998). Also upregulated were vacuolar iron transporters and a gene involved in zinc detoxification (PCR2), whereas various sugar metabolism genes were downregulated (Table S2).

**Figure 2:**
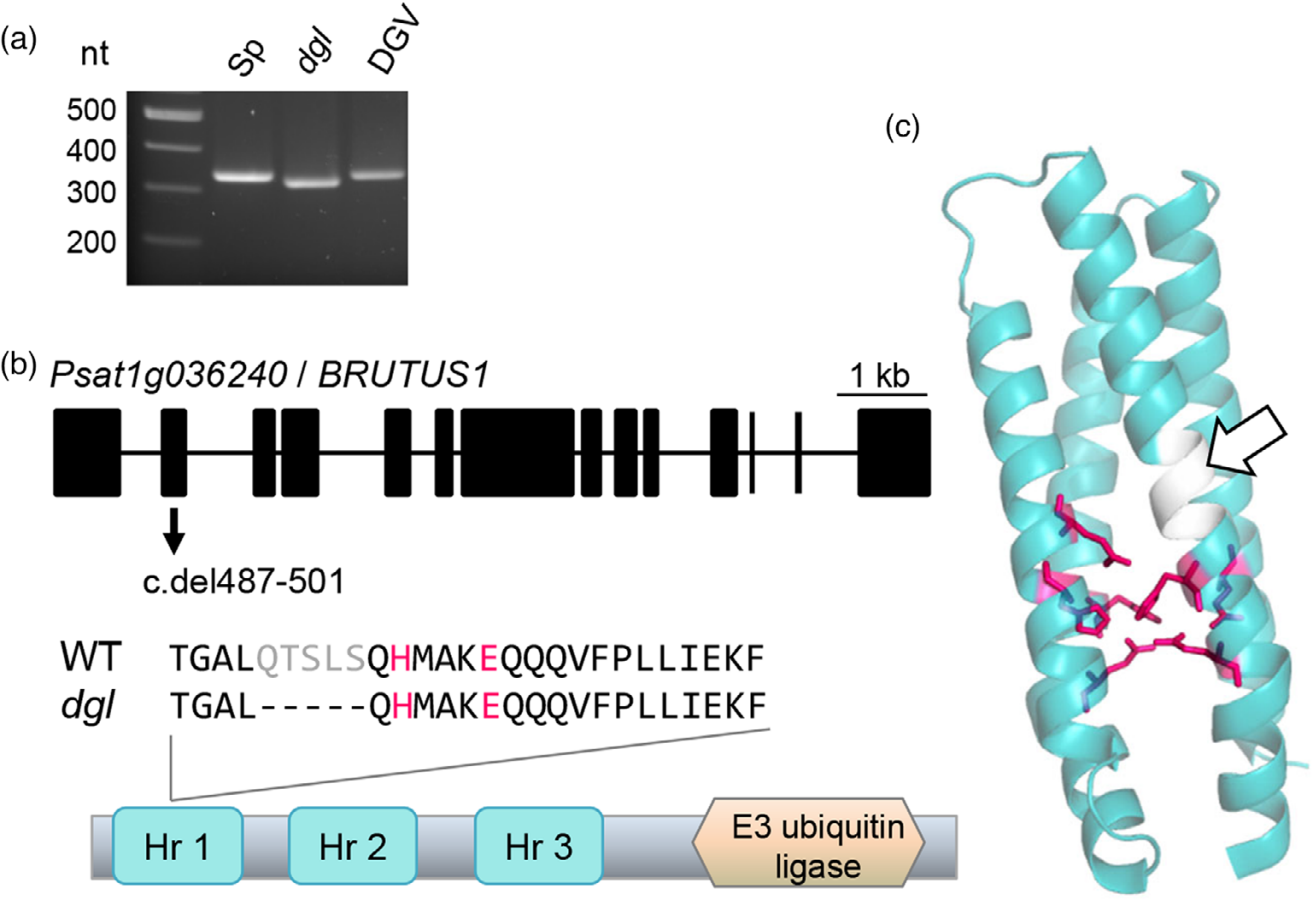
*dgl* is associated with a small in‐frame deletion in *BRUTUS*. (a) PCR analysis with primers spanning the 15‐bp deletion in *dgl* show that the deletion is absent from the near‐isogenic wild‐type Sparkle (Sp) variety and also from the Dippes Gelbe Viktoria (DGV) variety that was originally used for mutagenesis. Co‐segregation data is shown in Figure S2. (b) The *dgl* mutation is located in exon 2 of the gene *Psat1g036240*, encoding a BRUTUS homolog. The 15‐bp in‐frame deletion removes five amino acids (Q_163_TSLS) of the first hemerythrin domain, close to two iron‐binding residues (H and E, in red) conserved in hemerythrins. (c) Protein model of the Hr1 domain, with five of the seven ligands involved in binding of the di‐iron centre in magenta. The deleted 5 amino acids in the *dgl* mutant are rendered in white and marked with a white arrow. The deletion is predicted to displace two of the iron‐binding ligands, see Figure S3.

Alignment of the RNA reads to the pea genome sequence (Kreplak et al., 2019) followed by identification of SNPs and InDels in *dgl* relative to Sparkle confirmed that the lines were near‐isogenic (Figure S1). Because the mutant was generated by X‐rays, we focused on the InDels as candidate genetic polymorphisms (Table 1). PCR analysis showed that a deletion located on chromosome 1 co‐segregated invariably with the high‐iron phenotype in the F_2_ population (*n* = 11 mutants out of 44 plants, Figure S2) whereas the 3 InDels located on chromosome 6 were not linked to the mutant phenotype. The chromosome 1 InDel is not present in the original Dippes Gelbe Viktoria pea variety used for mutagenesis (Figure 2a). The 15‐bp deletion is located in exon 2 of *Psat1g036240*, corresponding to an in‐frame deletion of five amino acids (Figure 2b). A blastp search against the Arabidopsis proteome revealed that Psat1g036240 shares 66.4% identity with the BRUTUS (BTS) protein from Arabidopsis, an E3 ubiquitin ligase that acts as a negative regulator of iron uptake (Rodríguez‐Celma et al., 2019; Selote et al., 2015). The five deleted amino acids, QTSLS, are located in the first hemerythrin motif (Figure 2b) and are semi‐conserved in BTS sequences across the green lineage. Loss of these residues is predicted to displace two of the seven amino acid ligands that coordinate a diiron centre, thus potentially weakening iron binding and affecting the proposed oxygen and/or iron sensing function of this domain (Figure 2c; Figure S3). Alternatively, the deletion could affect the stability of the BTS protein.

**Table 1:** InDels identified by comparing leaf RNA‐seq data from *dgl* and wild‐type variety Sparkle mapped to the pea genome (variety Cameor). Only the deletion on chromosome 1 co‐segregated with the *dgl* phenotype (see Figure S2)

Chromosome/Linkage group	InDel size	Gene	Outcome of mutation	Arabidopsis BLAST hits	Co‐segregates?
Chr1/LG6	15 bp	*Psat1g036240*	In‐frame deletion of TSLSQ	BRUTUS	Yes
Chr6/LG2	24 bp	*Psat6g181240*	In‐frame deletion of SMQQPLPS	Decapping 5 (DCP5)	No
Chr6/LG2	15 bp	*Psat6g182800*	In‐frame deletion of QTLPL	Mediator‐associated protein 1‐like	No
Chr6/LG2	21 bp	*Psat6g183600*	Insertion causes premature stop, deleting final VLVQ	No hits	No

### *brz* is associated with a point mutation in *OPT3*

**Figure 3:**
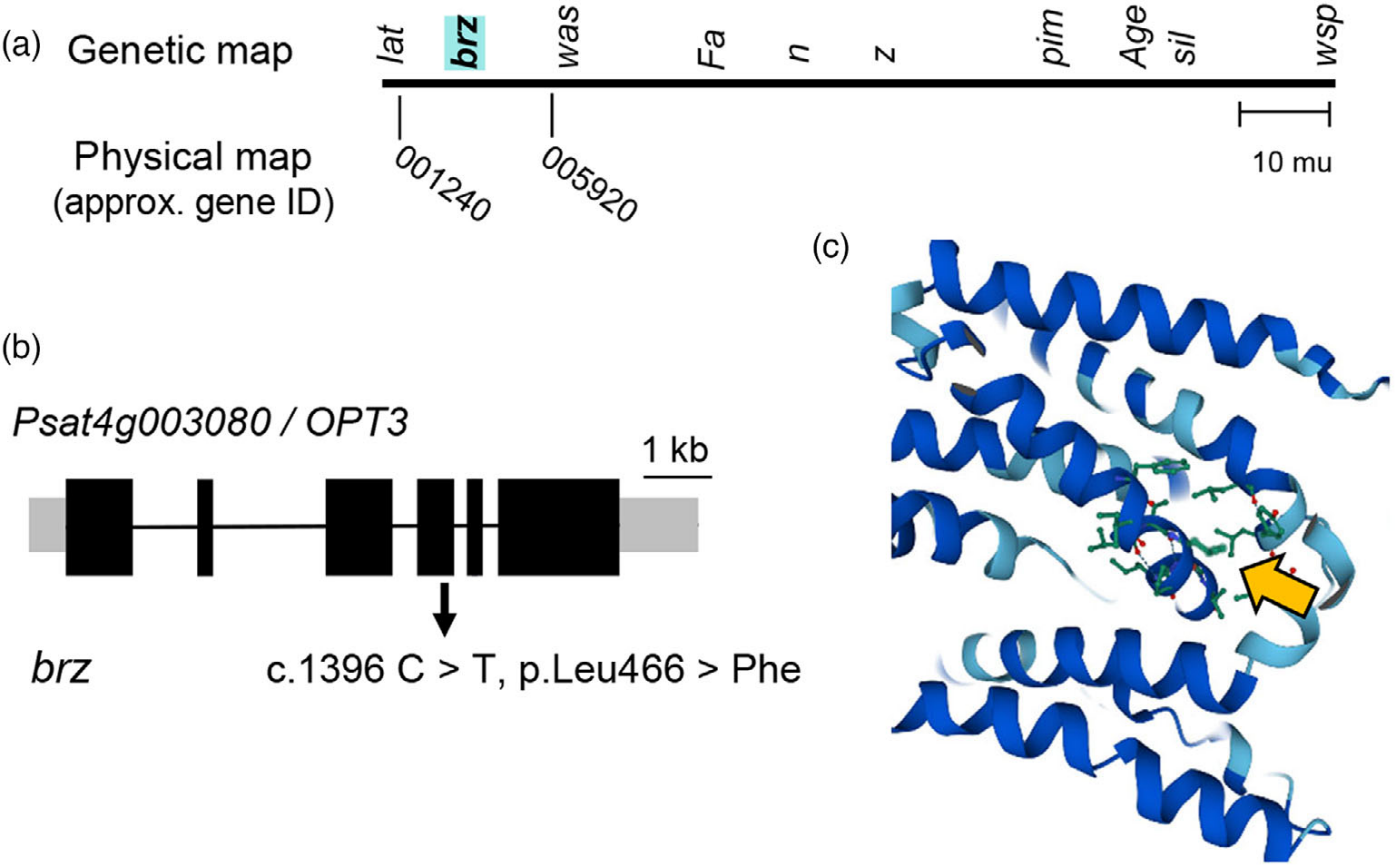
*brz* is associated with a point mutation in *OPT3*. (a) The *brz* mutation was previously mapped to the tip of chromosome 4, between the phenotypic loci *lat* and *was* (Ellis & Poyser, 2002; Kneen et al., 1990). Neigbouring loci that have been cloned were used to obtain a physical interval of ~450 genes. (b) The *brz* mutant contains a C to T mutation in exon 4 of the gene *Psat4g003080*, encoding the oligopeptide transporter OPT3, resulting in substitution of the highly conserved leucine 466 to phenylalanine. Co‐segregation data is shown in Figure S5. (c) AlphaFold2 model of Arabidopsis OPT3, centred on Leu461 (equivalent to Leu466 in pea OPT3, yellow arrow).

The *brz* mutation was previously mapped to the tip of chromosome 4 between the phenotypic markers *lat* (latum, Latin for wide leaves) and *was* (waxy stipules) (Ellis & Poyser, 2002; Kneen et al., 1990; Figure 3a). These two loci have not been identified, but closely linked genes that have been cloned suggested that *brz* lies between *Psat4g001240* and *Psat4g005920*, an interval of more than 450 genes. Near the middle of this interval are the likely gene candidates *Psat4g003000* and *Psat4g003080*, paralogous genes encoding the oligopeptide transporter OPT3. This member of the large OPT transporter family is unique to vascular plants and forms a separate phylogenetic clade with a single origin. Mutant studies in Arabidopsis showed that OPT3 has an essential function in transporting iron from the xylem to the phloem (Mendoza‐Cózatl et al., 2014; Zhai et al., 2014) and also mediates copper transport (Chia et al., 2023). Publicly available transcriptome data of pea suggests that *Psat4g003000* does not have meaningful expression levels whereas *Psat4g003080* is expressed in all plant organs, particularly in young leaves and stems (Figure S4b). Sequencing of the coding sequence identified a C to T mutation, consistent with the effect of EMS as a mutagen, that co‐segregated with iron accumulation in the leaves (Figure S5). The missense mutation changes a conserved leucine residue into phenylalanine (Leu466Phe, Figure 3b). Protein modeling suggests that Leu466 is part of an alpha helix oriented inwards (Figure 3c) and substitution to a larger, more hydrophobic side group may interfere with the transport mechanism.

### Genetic evidence for the mutations of *dgl* and *brz*

To confirm that the identified mutations are causally linked to the *dgl* and *brz* phenotypes, we used different approaches. First, genetic complementation in pea was attempted by transiently expressing the wild‐type pea *BTS1* cDNA in *dgl* seedlings, using *Agrobacterium*‐mediated leaf infiltration. This did not decrease or prevent iron accumulation in the infiltrated leaves, either because the expression was too low (GFP levels expressed using a separate construct were also low) or because iron accumulation in this mutant is controlled systemically and cannot be suppressed locally. Stable transformation of pea has been reported but is technically challenging. Therefore, as a second approach, we applied TILLING (Targeting Induced Local Lesions IN Genomes) to the syntenic Medicago genes. An EMS‐mutagenized population was screened for sequence polymorphisms in a 1.5–2 kb DNA region overlapping with or close to, respectively, the position of the *brz* and *dgl* mutations.

**Figure 4:**
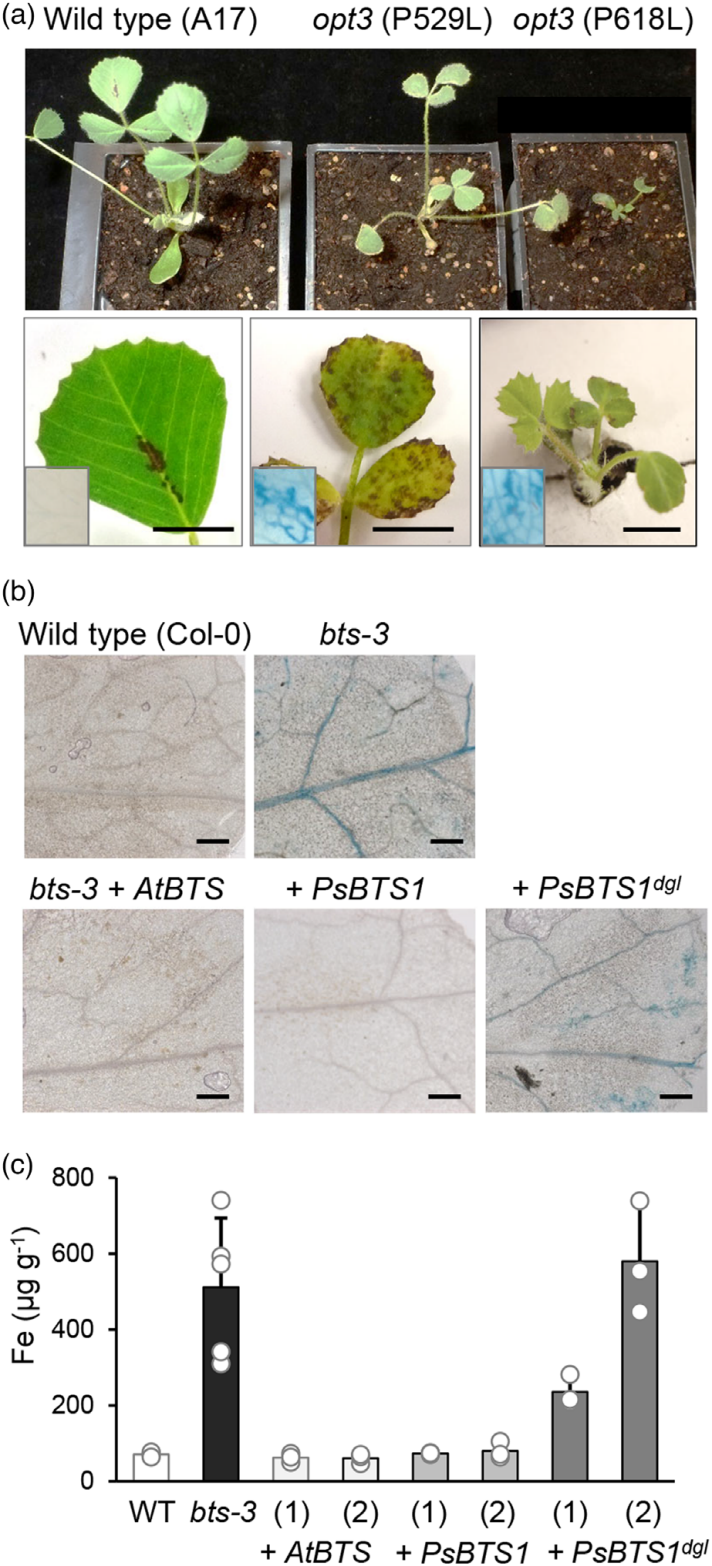
Genetic evidence for the mutated genes in *dgl* and *brz*. (a) Mutations in *Medicago truncatula OPT3*, which is syntenic with pea *OPT3*/*Psat4g003080*, phenocopy the pea *brz* mutant, including necrotic leaf spots and iron accumulation. Wild type (A17) and mutants in *OPT3* (*Medtr6g083900*), c.1586C>T (p.P529>L) and c.1853C>T (p.P618>L) as indicated, grown on soil for 3 weeks (top); close up of the leaves (middle); and leaf sectors stained for iron (insets). Scale bars are 0.5 cm. (b) Genetic complementation of the Arabidopsis *bts‐3* mutant with the wild‐type Arabidopsis coding sequence (AtBTS), wild‐type pea (*Ps*) *BTS1*, and the pea *BTS1*‐*dgl* sequence. Detail of rosette leaves stained for iron, imaged by light microscopy. Scale bars are 0.2 mm. See Figure S6 for genotyping data. (c) Iron concentration in rosette leaves of plants described in (b). For the transgenic plants, two independent lines in the T2 generation are shown. Bars represent the mean of 3–5 plants ± SD. *P* < 0.001 for *bts‐3* + *PsBTS1* ^*dgl*^ compared to wild‐type (WT), Student's *t*‐test.

For *OPT3*, we isolated four non‐synonymous mutations altering conserved amino acid residues (Table S3). Of those, Medicago plants with Pro529Leu or Pro618Leu presented with bronze spots on the leaves and intense iron staining (Figure 4a). Pro618Leu homozygous seedlings segregated at a low frequency and had a severe growth phenotype, confirming the critical function of *OPT3* in Medicago. For *BTS1*, an unusually small number of mutations was found in the selected region (exons 7 and 8, corresponding to the Hr 3 domain, Figure 2c). Of the five non‐synonymous mutations, only two affected conserved amino acids, Asp730 and Asp912. Asp912Asn had no discernible phenotype, whereas Asp730Asn homozygous plants could not be found among the offspring of a heterozygous plant. This suggests that *BTS1* is an essential gene in Medicago, similar to *BTS* being essential for embryo development in Arabidopsis (Selote et al., 2015), and prevented further studies in young seedlings or mature plants.

Because no other exome sequence polymorphisms were found in close range of the 15‐bp deletion associated with *dgl*, it is rather unlikely that a different, yet genetically linked, mutation causes the iron‐accumulating phenotype. Nevertheless, as a third approach, we tested for complementation of the Arabidopsis *bts‐3* mutant (Hindt et al., 2017) with the coding sequences of wild‐type pea *BTS1* or the *dgl*‐associated mutant gene. As control, plants were transformed with Arabidopsis *BTS*. A previous study showed that *bts‐3* mutant plants, which carry a point mutation in the E3 ligase domain, accumulated significant amounts of iron and had a severe growth defect (Hindt et al., 2017). We found that in the T2 generation growth was variable, perhaps because of epigenetic effects. However, iron accumulation visualized by Perls' staining, which was most prominent in the veins, was suppressed in *bts‐3* plants transformed with *AtBTS* or pea *BTS1*, but not with the *dgl*‐associated variant of *BTS1* (Figure 4b; Figure S6). Quantitative assays confirmed that *pea BTS1* expression reestablished normal leaf iron concentration in *bts‐3*. By contrast, the *BTS1*‐*dgl* gene could only partially or not at all suppress iron accumulation in two independent transgenic lines (Figure 4c). These results demonstrate that pea *BTS1* is a functional orthologue of Arabidopsis *BTS* and that the 15‐bp deletion is deleterious for BTS function.

### Explaining the phenotypes of *dgl* and *brz* through the respective mutant gene functions

**Figure 5:**
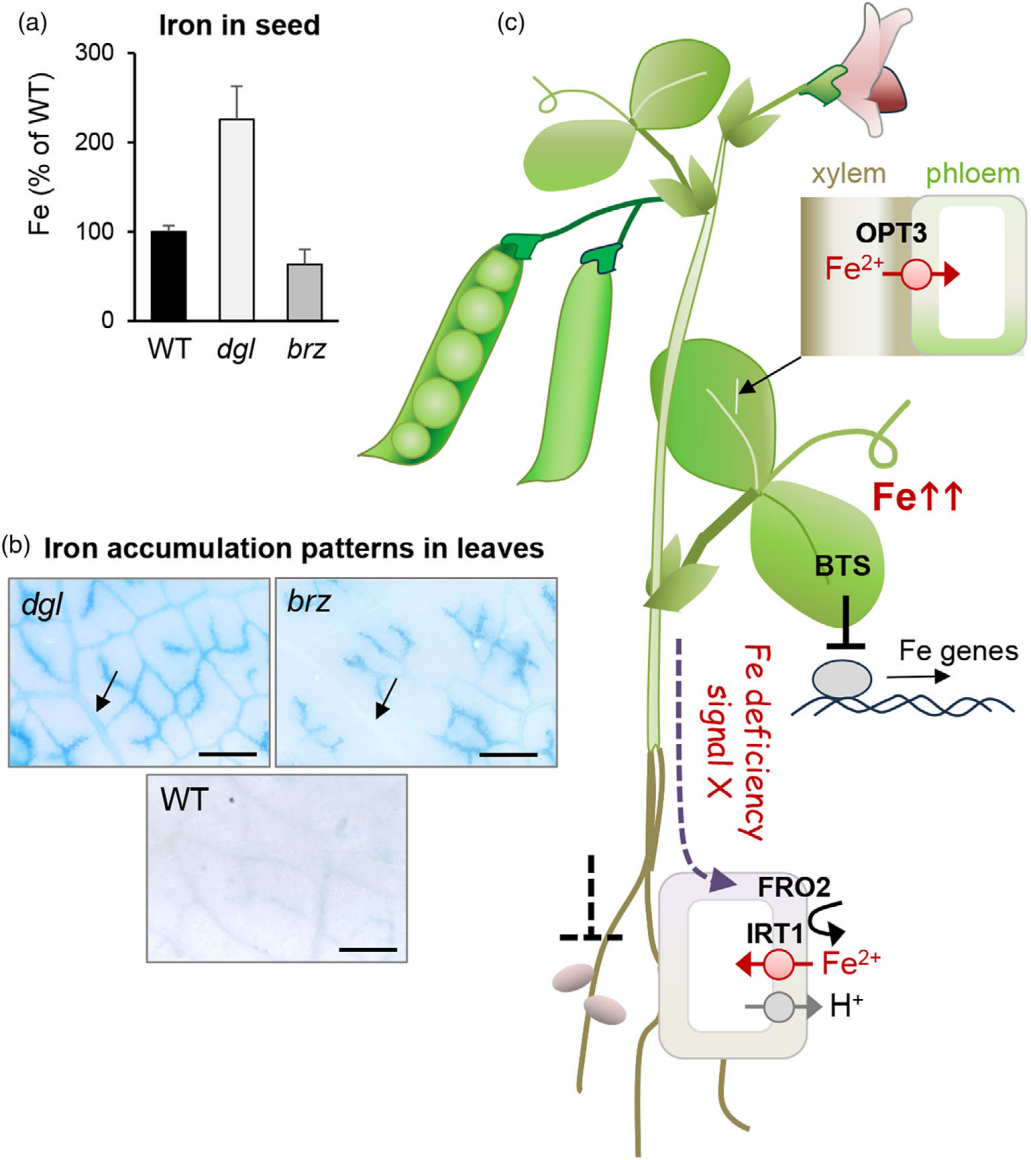
Integration of *dgl* (*bts1*) and *brz* (*opt3*) phenotypes with current knowledge of *BTS* and *OPT3* gene function. The *dgl* and *brz* mutants are similar in hyper‐accumulating iron in the leaves and deregulated iron uptake in the roots through a long‐distance signal as shown by previously reported grafting experiments. However, the mutants differ in (a) iron concentration in the seeds, measured by ICP‐OES and (b) the pattern of iron accumulation in the veins using Perls' reagent. Scale bar = 0.5 mm. (c) Proposed mechanisms underlying the phenotypes of the mutants, see main text for more details.

The pea *dgl* and *brz* mutants have an unusual history in that detailed reports of their phenotypes have been published since 1990, which can only now be matched to the affected genes. Similarities and differences in the mutant phenotypes can now be better understood in the context of what we know about the function of the BTS1 and OPT3 proteins, respectively (Figure 5). Both the *dgl* and *brz* pea mutants accumulate large amounts of iron in leaves, similar to *bts* and *opt3* mutants in Arabidopsis. This is a consequence of deregulated iron uptake in the roots: despite iron sufficiency, the roots of the pea mutants take up more iron, as shown using iron isotopes, facilitated by induced ferric chelate reductase activity and proton efflux (Grusak et al., 1990; Grusak & Pezeshgi, 1996; Welch & LaRue, 1990). To identify the *dgl* and *brz* mutations and validate them in other model organisms, we focussed on the leaf iron phenotype, since previous studies had shown that the leaf and root phenotypes were caused by the same genetic locus in each mutant (Grusak et al., 1990; Kneen et al., 1990; Welch & LaRue, 1990). Nevertheless, it would be interesting to assess the full phenotypic spectrum of the Medicago *opt3* mutants identified by TILLING, and in *bts1* and *bts2* mutants when these become available.

Aside from similarities, it is important to highlight the phenotypic differences in the *dgl* and *brz* mutants. As previously reported (Marentes & Grusak, 1998), *dgl* plants accumulate iron in seeds (Figure 5a). By contrast, hydroponically grown *brz* plants contained a similar amount of total seed iron as wild type (Grusak, 1994) and when we grew the plants on compost we found ~35% less iron in *brz* seeds compared to wild type (Figure 5a; Figure S7). The same changes in seed iron content were noted in the Arabidopsis *bts* and *opt3* mutants (Hindt et al., 2017; Selote et al., 2015; Stacey et al., 2008). Another phenotypic difference not previously reported is the different patterns of iron accumulation in the leaves: in the *brz*/*opt3* mutant, iron accumulated at the tips of minor leaf veins (Figure 5b). This is consistent with the high expression of the Arabidopsis *OPT3* gene in this location (Zhai et al., 2014). By contrast, the *dgl* mutant accumulated iron in both minor and major veins, matching the promoter activity of Arabidopsis *BTS* (Selote et al., 2015) which can reasonably be extrapolated to the pea *BTS1* gene.

Grafting experiments with the *dgl* and *brz* mutants first demonstrated the existence of a shoot‐to‐root signal for iron deficiency (Grusak & Pezeshgi, 1996; Welch & LaRue, 1990; Figure 5c). Similarly, grafting or wild‐type *OPT3* expression in leaves of the Arabidopsis *opt3‐2* mutant provided evidence for such a signal in Arabidopsis (Mendoza‐Cózatl et al., 2014; Zhai et al., 2014). These kinds of experiments remain to be carried out with Arabidopsis *bts* mutants and thus the pea grafting studies by Grusak and Pezeshgi (1996) are ‐ in hindsight ‐ evidence that BTS plays a role in systemic iron signaling. The molecular nature of the signal is unknown, but it is thought to be triggered by a low iron concentration in the phloem. Iron levels in phloem sap of the Arabidopsis *opt3‐2* mutant are ~50% lower than in the wild type (Zhai et al., 2014) which logically results in a poor supply of iron to the seeds. In the *dgl*/*bts1* mutant, seeds accumulate up to 4‐fold more iron, which is a magnitude less than the leaves. It is possible that iron accumulation in the leaf veins (by as yet unknown mechanisms) causes iron deficiency in the phloem. Impaired *bts1* function in the seeds then leads to a modest accumulation of iron drawn from the limited phloem supply. Measuring the phloem iron concentration in pea *dgl*/*bts1* or Arabidopsis *bts* mutants is required to confirm this idea and may also help to shed light on the puzzling findings that a *dgl* shoot could only be grafted on wild‐type roots as a side‐branch, and that removal of the cotyledons in a *dgl* mutant strongly increased root ferric chelate reductase activity (Grusak & Pezeshgi, 1996).

Interestingly, in grafting experiments, *brz* shoots also suppressed the number of symbiotic root nodules (Huynh & Guinel, 2020) and it is possible that the same signaling molecule leads to both physiological outputs. The role of neither BTS nor OPT3 in iron homeostasis during nodule development has been properly investigated (Day & Smith, 2021). *opt3* mutants will be invaluable to shed light on the question of whether iron is delivered to nodules via the xylem or phloem, or both. *bts* mutants could help lift the lid on the regulation of iron homeostasis in nodules, including downstream transcription factors.

Increasing the mineral micronutrient of crops, or biofortification, is an important area of research to combat hidden hunger and to provide nutritious plant‐based diets in the face of climate change. The identified mutations indicate that either BTS or OPT3 function could be modified to increase the iron content of vegetative tissues. Genetic variants of *BTS* could also enhance iron in seeds. However, there is a fine balance between increasing iron and toxicity symptoms, and null mutants of *BTS* and *OPT3* are embryo‐lethal (Mendoza‐Cózatl et al., 2014; Selote et al., 2015; Zhai et al., 2014). Interestingly, the pea *dgl* mutant has relatively minor growth defects, indicating that it is worth screening for weaker alleles, especially in the Hr1 domain. In summary, knowing the causal mutations in the historic *dgl* and *brz* mutants will help to further unravel the functional roles of these important iron homeostasis genes.

## EXPERIMENTAL PROCEDURES

### Plant material and growth

Seeds of *dgl* (JI3085) and the wild type ‘Sparkle’ (JI0427) were obtained from the John Innes Centre Germplasm Resource Unit (GRU), which were donated to the collection by Michael Grusak, then at Asgrow Seeds, Twin Falls, ID, USA. Seeds of the EMS mutant E107, also named *brz* (JI2616) were also obtained from GRU, donated by Thomas LaRue, Cornell University, Ithaca, USA. The germination rate of *brz* seeds declines rapidly over time, and the mutant should be propagated every 3–4 years. The *dgl* mutant was originally generated in the pea variety Dippes Gelbe Viktoria (DGV, accession JI2413 in the GRU collection). Marentes and Grusak (1998) backcrossed *dgl* at least five times with the Sparkle variety, selected at the F_3_ stage for the high‐iron phenotype. For segregation analysis, *dgl* and *brz* were crossed with line JI804 because of its contrasting phenotypic traits and suitable genetic markers. Plants were germinated on peat‐based compost (Levington F2) and grown in a greenhouse with additional lighting in the winter and watering as required.

### Leaf iron staining and quantification

Leaf samples were stained for iron using Perls' reagent as previously described (Meguro et al., 2007). Samples were mounted in glycerol (50% v/v) and imaged on an Axio Zoom.V16 stereo microscope with an Axiocam 512 color camera (Zeiss, Cambourne, UK). For measuring iron concentrations, dried leaf samples were digested in 0.25 ml nitric acid (69% w/v) and 0.25 ml hydrogen peroxide (30% w/v) at 90°C. After neutralizing with 1 ml ammonium acetate (15% w/v), samples were reduced with 0.1 ml ascorbic acid (4% w/v). Fe^2+^ was quantified using the colorimetric iron chelator ferene (3‐(2‐pyridyl)‐5,6‐bis‐[2‐(5‐furyl‐sulfonic acid)]‐1,2,4‐triazine, 0.1% w/v) and absorbance was measurement at 593 nm. Iron and other elements in pea seeds were quantified by Inductively Coupled Plasma‐Optical Emission Spectroscopy following digestion of ground samples in nitric acid (55% w/v) and hydrogen peroxide (6% w/w) at 95°C for 16 h.

### RNA extraction and Illumina sequencing

Leaf tissue was sampled from three plants of each genotype (Sparkle and *dgl*) and snap‐frozen in liquid N_2_. The frozen tissue was ground to a fine powder before RNA extraction using TRIzol® Reagent (ThermoFisher, Basingstoke, UK) and DNase treatment with TURBO DNase (ThermoFisher). The quality and quantity of RNA were verified with the Agilent Bioanalyzer RNA 6000 Nano assay before cDNA library preparation (250–300 bp insert) and Illumina Sequencing (PE 150, Novogene, Cambridge, UK).

### Differential expression analysis

Illumina reads were pseudo‐aligned against the *P. sativum* reference transcriptome (Kreplak et al., 2019) using Kallisto (Bray et al., 2016). Gene expression levels were determined using the R package Sleuth (Pimentel et al., 2017) using the Wald test, where we considered genes to be differentially expressed between genotypes with *q* < 0.05. Enrichment of GO terms was calculated using the R package goseq (Young et al., 2010).

### Identification of deletions

Illumina reads were aligned against the *P. sativum* reference genome (Kreplak et al., 2019) using the software *BWA‐mem* (Li, 2013). The software transIndel (Yang et al., 2018) was then used to identify the location of deletions and insertions using the default parameters except for DP = 1 (to capture all possible deletions, irrespective of gene expression level).

### PCR analysis to confirm allelic variation

DNA was extracted from individual F_2_ plants in 200 mm Tris–HCl pH 7.5, 250 mm NaCl, 25 mm EDTA, and 0.5% (w/v) sodium dodecyl sulfate and precipitated with 50% (v/v) isopropanol. For *dgl*, PCR primers were designed to amplify across the region of *Psat1g036240* which contains the identified five codon deletion: GCGTGAAGAATGTAGCACAG and ACCTGCAATATTCAACCAGCA, see Table S4. Amplified fragments were run on a 2% (w/v) agarose gel for 2 h at 70 V, allowing separation of the wild‐type Sparkle band (334 bp) and the *dgl* band (319 bp). For *brz*, PCR primers were designed to span part of the *Psat4g003080* coding sequence: GACATATTGAGACAGAGCAGG and ATACCGAATCATGAACTGTGC, see Table S4. The PCR product was purified and sequenced.

### Protein homology modeling

The first hemerythrin domain of pea BTS1/Psat1g036240.1 (amino acids 55–184), the *dgl* variant of this domain, the full‐length OPT3 protein and the L466F variant were modeled using AlphaFold2 and visualized using PyMol version 2.5.2.

### Genetic complementation of Arabidopsis *bts‐3*

Heterozygous *bts‐3* plants (Hindt et al., 2017) were transformed with plasmid pICSL869550OD (SynBio, Norwich, UK) carrying either the Arabidopsis *BRUTUS* coding sequence (*BTS*, *AT3G18290*); the pea *BRUTUS1* coding sequence (*Psat1g036240*); or the pea *dgl* variant of *BRUTUS1* (lacking nucleotides 487–510). The coding sequences were placed downstream of the Arabidopsis *BRUTUS* promoter, nucleotides −1904 to −1, and upstream of the *ocs* terminator, using Golden Gate assembly. All constructs were verified by sequencing. T1 plants homozygous for *bts‐3* were selected by PCR (see Table S4 for primers) followed by restriction with PflMI, the recognition site of which is deleted in the *dgl* allele. Of these lines, T2 plants carrying the transgene were scored for growth and iron accumulation.

### *Medicago truncatula* TILLING

An M2 population of EMS‐mutagenized *Medicago truncatula* was screened for genetic polymorphisms in *BTS1*/*Psat1g036240* and *OPT3*/*Psat4g003080*. For *BTS1*, primers MtBTS1‐F1 and ‐R spanned exons 7–10 to maximize the ratio of exon: intron sequence; For *OPT3*, primers MtOPT3‐F1 and ‐R3 spanned exons 4–6, surrounding the *brz* mutation. See Table S4 for primer sequences. For the selected lines (Table S4), seedlings were grown up and inspected for phenotypes and iron accumulation using Perls' staining.

## Supporting information


**Figure S1.** Exome mapping to identify the *dgl* mutation. Circular representation of the pea (*Pisum sativum* L.) genome to which the RNA‐seq data from *dgl* and Sparkle (wild type) leaves are mapped. Blue lines represent sequence polymorphisms and yellow indicates sequence identity between *dgl* and Sparkle.
**Figure S2.** Co‐segregation analysis of the *dgl* mutation and iron accumulation in pea, *Pisum sativum* L.
(a) The *dgl* mutant was crossed with pea accession JI804, acting as wild‐type for the locus, to obtain an F_2_ population of 44 plants.
(b) Discs (3 mm diameter) of the second complete leaf were stained for iron and scored for the iron‐accumulating phenotype.
(c) PCR analysis of F_2_ plants (representative selection), *dgl* and the wild‐type control Sparkle (Sp), to detect the 15‐bp deletion in *Psat1g036240* as well as the wild‐type allele.
**Figure S3.** Modelling of the amino acid ligands of the diiron centre in the hemerythrin 1 domain, in wild‐type pea BRUTUS (left) and in the *dgl* variant protein (right). The diiron centre is predicted to have 7 ligands (plus water or oxygen) following a pattern that is conserved in hemerythrins (H…HxxxE…H…HxxxE), consisting of histidine (His, H), glutamate (Glu, E) plus one glutamine (Gln, Q). Because of the 5 amino acid deletion in the *dgl* mutant, the nearby His169 and Glu173 are predicted to be displaced and pointing away from the active site. This may affect iron binding or protein stability.
**Figure S4.** The pea homolog *OPT3* is a candidate gene for *BRZ*.
(a) The *brz* mutation was previously mapped to the tip of chromosome 4, between the genetic markers *lat* and *was* (Kneen et al., 1990; Ellis & Poyser, 2002).
(b) Detail of chromosome 4 showing the two neighboring *OPT3* paralogs, *Psat1g003000* and *Psat1g003080*. Expression data in transcripts per million (TPM) from https://urgi.versailles.inra.fr/ indicate that only one of the two paralogs, *Psat1g003080*, is expressed.
**Figure S5.** Co‐segregation analysis of the *brz* mutation and iron accumulation in pea, *Pisum sativum* L.
(a) Iron‐stained leaf discs (3 mm diameter) of F_2_ plants from crosses between *brz* and JI804 used as wild type. The F_2_ were from 3 different F_1_ plants.
(b) Allele variation for c.1396 in *Psat4g003080*/*PsOPT3* in 14 F_2_ plants, wild‐type Sparkle (Sp) and *brz* serving as negative and positive controls, respectively.
**Figure S6.** Genotyping of the plants pictured in Figure 4b. The *bts‐3* mutation removes a PflMI restriction site which can be detected by PCR followed by PflMI digestion. Sizes of the nucleotide bands are on the right. Primers are listed in Table S4.
**Figure S7.** Iron and zinc concentrations in pea seeds of *brz* compared to wild type. Seeds were collected from plants grown on compost. Iron (Fe), zinc (Zn), manganese (Mn) and phosphorus (P) were measured by Inductively Coupled Plasma‐Optical Emission Spectroscopy. Values are the mean ± SD of representative seeds from 5 different plants. **P < 0.01, Student *t*‐test.
**Table S1.** Gene Ontology terms of differentially expressed genes in leaves from the pea *dgl* mutant compared to the corresponding wild‐type variety Sparkle. BP, biochemical pathway; MF, molecular function.
**Table S2.** Differentially expressed genes in leaves from the pea *dgl* mutant compared to the corresponding wild‐type variety Sparkle.
**Table S3.** TILLING mutations in *Medicago truncatula* genes.
**Table S4.** Primers used in this study.

## References

[tpj16514-bib-0002] Becker, R. , Manteu, R. & Neumann, D. (1998) Excessive iron accumulation in the pea mutants *dgl* and *brz*: subcellular localization of iron and ferritin. Planta, 207, 217–223.

[tpj16514-bib-0003] Bray, N. , Pimentel, H. , Melsted, P. & Pachter, L. (2016) Near‐optimal probabilistic RNA‐seq quantification. Nature Biotechnology, 34, 525–527. 10.1038/nbt.3519 27043002

[tpj16514-bib-0004] Chia, J. , Yan, J. , Rahmati Ishka, M. , Faulkner, M. , Simons, E. , Huang, R. et al. (2023) Loss of OPT3 function decreases phloem copper levels and impairs crosstalk between copper and iron homeostasis and shoot‐to‐root signaling in *Arabidopsis thaliana* . Plant Cell, 35, 2157–2185. 36814393 10.1093/plcell/koad053 PMC10226573

[tpj16514-bib-0005] Connorton, J.M. & Balk, J. (2019) Iron biofortification of staple crops: lessons and challenges in plant genetics. Plant & Cell Physiology, 60, 1447–1456. 31058958 10.1093/pcp/pcz079 PMC6619672

[tpj16514-bib-0006] Day, D.A. & Smith, P.M.C. (2021) Iron transport across symbiotic membranes of nitrogen‐fixing legumes. International Journal of Molecular Sciences, 22, 432. 33406726 10.3390/ijms22010432 PMC7794740

[tpj16514-bib-0007] Ellis, T.H.N. , Hofer, J.M.I. , Timmerman‐Vaughan, G.M. , Coyne, C.J. & Hellens, R.P. (2011) Mendel, 150 years on. Trends in Plant Science, 16, 590–596. 21775188 10.1016/j.tplants.2011.06.006

[tpj16514-bib-0008] Ellis, T.H.N. & Poyser, S.J. (2002) An integrated and comparative view of pea genetic and cytogenetic maps. The New Phytologist, 153, 17–25.

[tpj16514-bib-0009] García, M.J. , Romera, F.J. , Stacey, M.G. , Stacey, G. , Villar, E. , Alcántara, E. et al. (2013) Shoot to root communication is necessary to control the expression of iron‐acquisition genes in strategy I plants. Planta, 237, 65–75. 22983673 10.1007/s00425-012-1757-0

[tpj16514-bib-0010] Gottschalk, W. (1987) Improvement of the selection value gene *dgl* through recombination. Pisum Newsletter, 19, 9–11.

[tpj16514-bib-0011] Grusak, M.A. (1994) Iron transport to developing ovules of *Pisum sativum*. I. Seed import characteristics and phloem iron‐loading capacity of source regions. Plant Physiology, 104, 649–655. 12232115 10.1104/pp.104.2.649 PMC159243

[tpj16514-bib-0012] Grusak, M.A. & Pezeshgi, S. (1996) Shoot‐to‐root signal transmission regulates root Fe(III) reductase activity in the *dgl* mutant of pea. Plant Physiology, 110, 329–334. 12226184 10.1104/pp.110.1.329 PMC157724

[tpj16514-bib-0013] Grusak, M.A. , Welch, R.M. & Kochian, L.V. (1990) Physiological characterization of a single‐gene mutant of *Pisum sativum* exhibiting excess iron accumulation: I. Root iron reduction and iron uptake. Plant Physiology, 93, 976–981. 16667609 10.1104/pp.93.3.976 PMC1062617

[tpj16514-bib-0014] Hindt, M.N. , Akmakjian, G.Z. , Pivarski, K.L. , Punshon, T. , Baxter, I. , Salt, D.E. et al. (2017) *BRUTUS* and its paralogs, *BTS LIKE1* and *BTS LIKE2*, encode important negative regulators of the iron deficiency response in *Arabidopsis thaliana* . Metallomics, 9, 876–890. 28620661 10.1039/c7mt00152e PMC5558852

[tpj16514-bib-0015] Huynh, C.A. & Guinel, F.C. (2020) Shoot extracts from two low nodulation mutants significantly reduce nodule number in pea. Plants, 9, 1–13. 10.3390/plants9111505 PMC7694783 33172149

[tpj16514-bib-0016] Kneen, B.E. , LaRue, T.A. , Welch, R.M. & Weeden, N.F. (1990) Pleiotropic effects of *brz* . Plant Physiology, 93, 717–722. 16667528 10.1104/pp.93.2.717 PMC1062575

[tpj16514-bib-0017] Kreplak, J. , Madoui, M.A. , Cápal, P. , Novák, P. , Labadie, K. , Aubert, G. et al. (2019) A reference genome for pea provides insight into legume genome evolution. Nature Genetics, 51, 1411–1422. 31477930 10.1038/s41588-019-0480-1

[tpj16514-bib-0018] Lahner, B. , Gong, J. , Mahmoudian, M. , Smith, E.L. , Abid, K.B. , Rogers, E.E. et al. (2003) Genomic scale profiling of nutrient and trace elements in *Arabidopsis thaliana* . Nature Biotechnology, 21, 1215–1221. 10.1038/nbt865 12949535

[tpj16514-bib-0019] Li, H. (2013) Aligning sequence reads, clone sequences and assembly contigs with BWA‐MEM. *arXiv*, 1303.3997.

[tpj16514-bib-0020] Marentes, E. & Grusak, M.A. (1998) Iron transport and storage within the seed coat and embryo of developing seeds of pea (*Pisum sativum* L.). Seed Science Research, 8, 367–375.

[tpj16514-bib-0021] Meguro, R. , Asoano, Y. , Odagiri, S. , Li, C. , Iwatsuki, H. & Shoumura, K. (2007) Nonheme‐iron histochemistry for light and electron microscopy: a historical, theoretical and technical review. Archives of Histology and Cytology, 70, 1–19. 17558140 10.1679/aohc.70.1

[tpj16514-bib-0022] Mendoza‐Cózatl, D.G. , Xie, Q. , Akmakjian, G.Z. , Jobe, T.O. , Patel, A. , Stacey, M.G. et al. (2014) OPT3 is a component of the iron‐signaling network between leaves and roots and misregulation of OPT3 leads to an over‐accumulation of cadmium in seeds. Molecular Plant, 7, 1455–1469. 24880337 10.1093/mp/ssu067 PMC4153440

[tpj16514-bib-0023] Pimentel, H. , Bray, N. , Puente, S. , Melsted, P. & Pachter, L. (2017) Differential analysis of RNA‐seq incorporating quantification uncertainty. Nature Methods, 14, 687–690. 28581496 10.1038/nmeth.4324

[tpj16514-bib-0024] Robinson, N. , Proctor, C. , Connolly, E. & Guerinot, M. (1999) A ferric‐chelate reductase for iron uptake from soils. Nature, 397, 694–697. 10067892 10.1038/17800

[tpj16514-bib-0025] Rodríguez‐Celma, J. , Chou, H. , Kobayashi, T. , Long, T.A. & Balk, J. (2019) Hemerythrin E3 ubiquitin ligases as negative regulators of iron homeostasis in plants. Frontiers in Plant Science, 10, 98. 30815004 10.3389/fpls.2019.00098 PMC6381054

[tpj16514-bib-0026] Selote, D. , Samira, R. , Matthiadis, A. , Gillikin, J.W. & Long, T.A. (2015) Iron‐binding E3 ligase mediates iron response in plants by targeting basic Helix‐Loop‐Helix transcription factors. Plant Physiology, 167, 273–286. 25452667 10.1104/pp.114.250837 PMC4281009

[tpj16514-bib-0027] Stacey, M.G. , Patel, M. , McClain, W.E. , Mathieu, M. , Remley, M. , Rogers, E.E. et al. (2008) The Arabidopsis AtOPT3 protein functions in metal homeostasis and movement of iron to developing seeds. Plant Physiology, 146, 589–601. 18083798 10.1104/pp.107.108183 PMC2245856

[tpj16514-bib-0028] Welch, R.M. & LaRue, T.A. (1990) Physiological characteristics of Fe accumulation in the ‘bronze’ mutant of *Pisum sativum* L., cv ‘Sparkle’ E107 (*brz brz*). Plant Physiology, 93, 723–729. 16667529 10.1104/pp.93.2.723 PMC1062576

[tpj16514-bib-0029] Yang, R. , Van Etten, J.L. & Dehm, S.M. (2018) Indel detection from DNA and RNA sequencing data with transIndel. BMC Genomics, 19, 270. 29673323 10.1186/s12864-018-4671-4 PMC5909256

[tpj16514-bib-0030] Young, M. , Wakefield, M. , Smyth, G. & Oshlack, A. (2010) Gene ontology analysis for RNA‐seq: accounting for selection bias. Genome Biology, 11, R14. 20132535 10.1186/gb-2010-11-2-r14 PMC2872874

[tpj16514-bib-0031] Zhai, Z. , Gayomba, S.R. , Il, J.H. , Vimalakumari, N.K. , Piñeros, M. , Craft, E. et al. (2014) OPT3 is a phloem‐specific iron transporter that is essential for systemic iron signaling and redistribution of iron and cadmium in Arabidopsis. Plant Cell, 26, 2249–2264. 24867923 10.1105/tpc.114.123737 PMC4079381

