# Supplementary material for: Genetic basis of the historical iron‐accumulating dgl and brz mutants in pea
Source: Plant J. 2023 Oct 26;117(2):590–8. doi: 10.1111/tpj.16514 (PMC10952674; doi:10.1111/tpj.16514)
Supplement: Supplementary file 1 — Figure S1. Exome mapping to identify the dgl mutation. Circular representation of the pea (Pisum sativum L.) genome to which the RNA‐seq data from dgl and Sparkle (wild type) leaves are mapped. Blue lines represent sequence polymorphisms and yellow indicates sequence identity between dgl and Sparkle. Figure S2. Co‐segregation analysis of the dgl mutation and iron accumulation in pea, Pisum sativum L. (a) The dgl mutant was crossed with pea accession JI804, acting as wild‐type for the locus, to obtain an F2 population of 44 plants. (b) Discs (3 mm diameter) of the second complete leaf were stained for iron and scored for the iron‐accumulating phenotype. (c) PCR analysis of F2 plants (representative selection), dgl and the wild‐type control Sparkle (Sp), to detect the 15‐bp deletion in Psat1g036240 as well as the wild‐type allele. Figure S3. Modelling of the amino acid ligands of the diiron centre in the hemerythrin 1 domain, in wild‐type pea BRUTUS (left) and in the dgl variant protein (right). The diiron centre is predicted to have 7 ligands (plus water or oxygen) following a pattern that is conserved in hemerythrins (H…HxxxE…H…HxxxE), consisting of histidine (His, H), glutamate (Glu, E) plus one glutamine (Gln, Q). Because of the 5 amino acid deletion in the dgl mutant, the nearby His169 and Glu173 are predicted to be displaced and pointing away from the active site. This may affect iron binding or protein stability. Figure S4. The pea homolog OPT3 is a candidate gene for BRZ. (a) The brz mutation was previously mapped to the tip of chromosome 4, between the genetic markers lat and was (Kneen et al., 1990; Ellis & Poyser, 2002). (b) Detail of chromosome 4 showing the two neighboring OPT3 paralogs, Psat1g003000 and Psat1g003080. Expression data in transcripts per million (TPM) from https://urgi.versailles.inra.fr/ indicate that only one of the two paralogs, Psat1g003080, is expressed. Figure S5. Co‐segregation analysis of the brz mutation and iron accumulation in pe [file TPJ-117-590-s001.pdf]

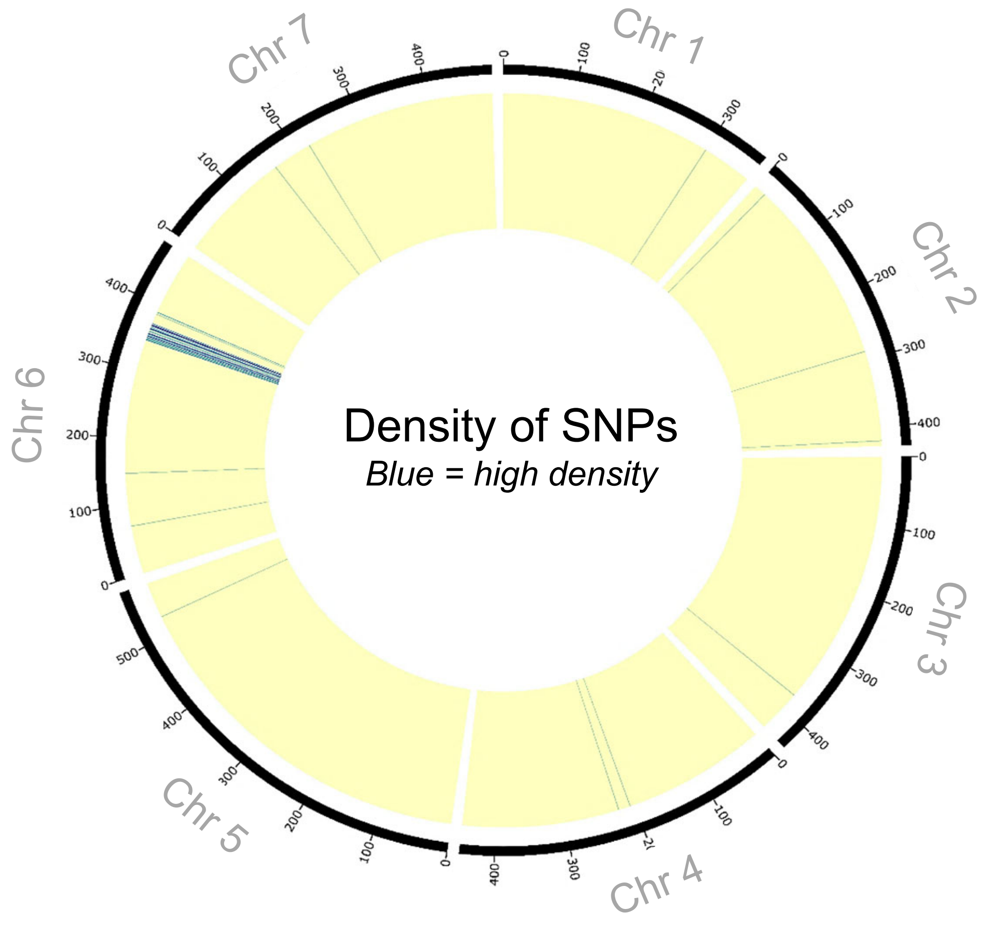

Supplemental Figure S1. Exome mapping to identify the *dgl* mutation.
Circular representation of the pea (*Pisum sativum* L.) genome to which the RNA-seq data from *dgl* and Sparkle (wild type) leaves are mapped. Blue lines represent sequence polymorphisms and yellow indicates sequence identity between *dgl* and Sparkle.

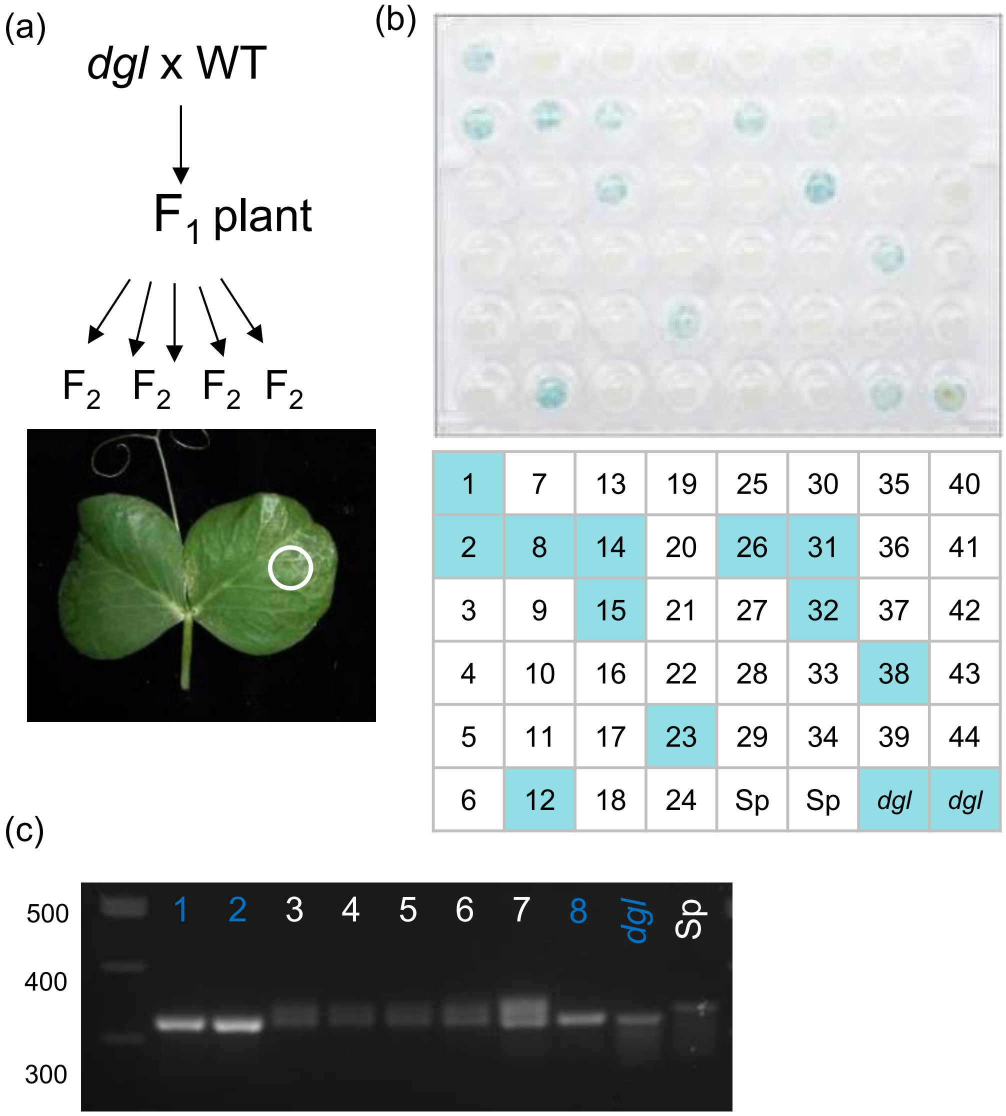

Supplemental Figure S2. Co-segregation analysis of the *dgl* mutation and iron accumulation in pea, *Pisum sativum* L.
(a) The *dgl* mutant was crossed with pea accession JI804, acting as wild-type for the locus, to obtain an F_2 population of 44 plants.
(b) Discs (3 mm diameter) of the second complete leaf were stained for iron and scored for the iron-accumulating phenotype.
(c) PCR analysis of F_2 plants (representative selection), *dgl* and the wild-type control Sparkle (Sp), to detect the 15-bp deletion in *Psat1g036240* as well as the wild-type allele.

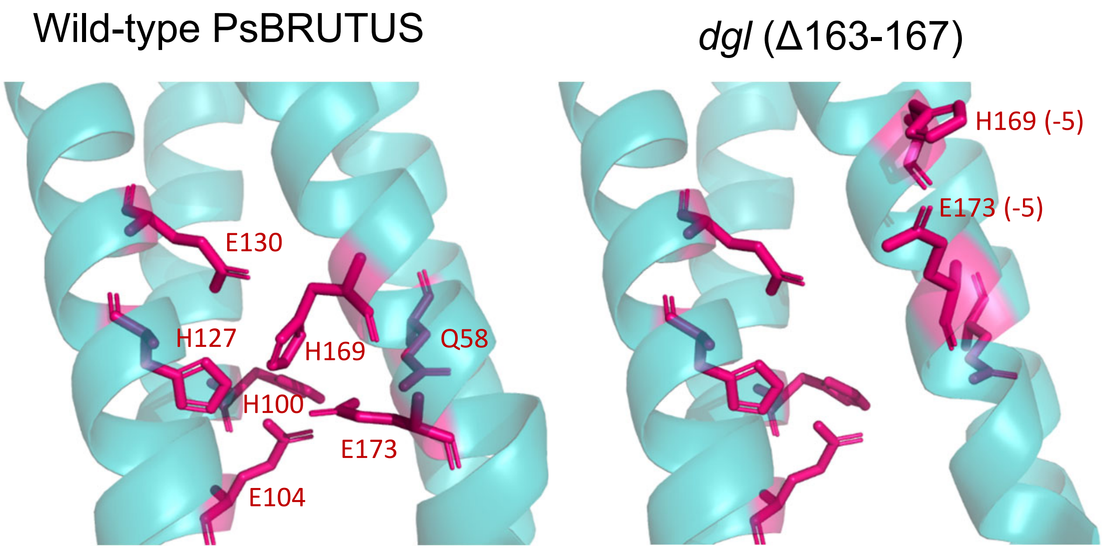

Supplemental Figure S3. Modelling of the amino acid ligands of the diiron centre in the hemerythrin 1 domain, in wild-type pea BRUTUS (left) and in the *dgl* variant protein (right). The diiron centre is predicted to have 7 ligands (plus water or oxygen) following a pattern that is conserved in hemerythrins (H…HxxxE…H…HxxxE), consisting of histidine (His, H), glutamate (Glu, E) plus one glutamine (Gln, Q). Because of the 5 amino acid deletion in the *dgl* mutant, the nearby His169 and Glu173 are predicted to be displaced and pointing away from the active site. This may affect iron binding or protein stability.

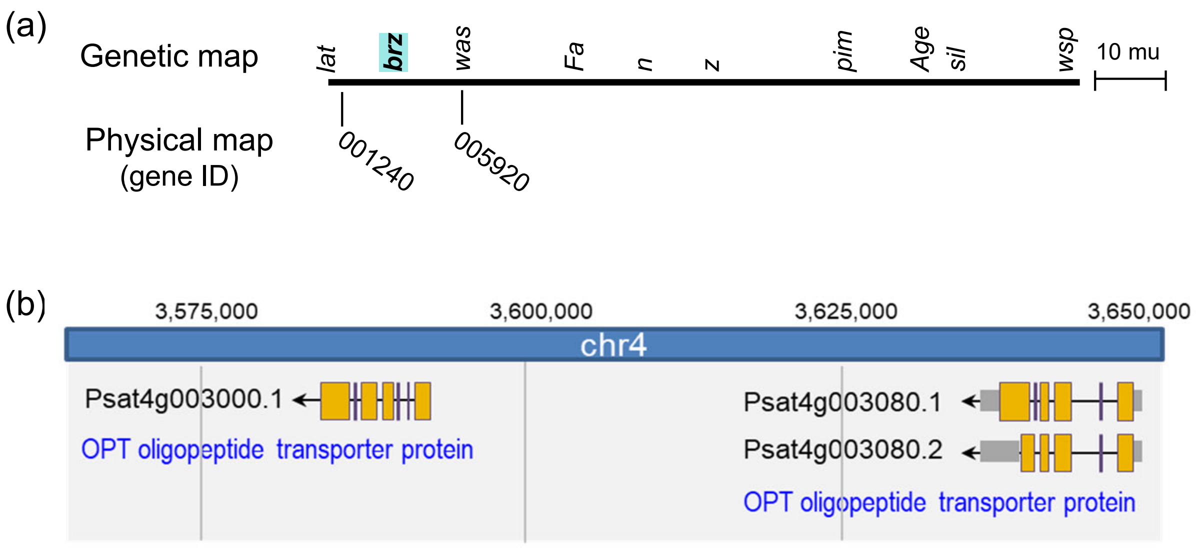

Expression levels (TPM)

	Root	Nodule	Stem	Lower leaf	Upper leaf	Flowers	Pods	Young seeds (12 dap)
Psat4g00**3000**	0	0	0.01	0	0.01	0	0.01	0
Psat4g00**3080**	3.39	9.08	19.44	16.63	28.58	12.35	3.49	12.29

https://urgi.versailles.inra.fr/download/pea/Pea_PSCAM_transcriptome/

Supplemental Figure S4. The pea homolog *OPT3* is a candidate gene for *BRZ.*
(a) The *brz* mutation was previously mapped to the tip of chromosome 4, between the genetic markers *lat* and *was* (Kneen et al., 1990; Ellis & Poyser, 2002).
(b) Detail of chromosome 4 showing the two neighboring *OPT3* paralogs, *Psat1g003000* and *Psat1g003080*. Expression data in transcripts per million (TPM) from https://urgi.versailles.inra.fr/ indicate that only one of the two paralogs, *Psat1g003080*, is expressed.

(a)

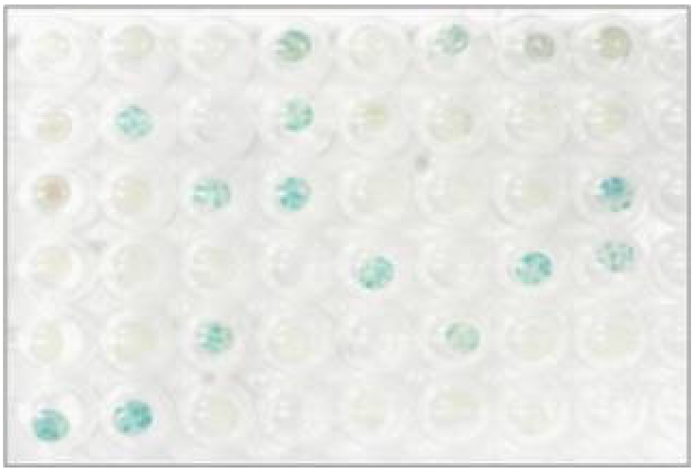

1-1	1-7	1-13	2-5	2-11	3-3	3-9	Sp
1-2	1-8		2-6	2-12	3-4	3-10	Sp
1-3	1-9	2-1	2-7	2-13	3-5	3-11	*brz*
1-4	1-10	2-2		2-14		3-12	*brz*
1-5	1-11	2-3	2-9		3-7	3-13	JI804
1-6	1-12	2-4	2-10	3-2	3-8	3-14	

(b)

	PsOPT3, position c.1396
1-2	C
1-3	C/T
1-4	C/T
1-5	C/T
1-6	T
1-7	C
1-8	T
1-12	T
2-2	C
2-5	T
2-7	T
2-9	C/T
2-12	C/T
3-3	T
Sp	C
brz	T

Supplemental Figure S5. Co-segregation analysis of the *brz* mutation and iron accumulation in pea, *Pisum sativum* L.
(a) Iron-stained leaf discs (3 mm diameter) of F_2 plants from crosses between *brz* and JI804 used as wild type. The F_2 were from 3 different F_1 plants.
(b) Allele variation for c.1396 in *Psat4g003080* / *PsOPT3* in 14 F_2 plants, wild-type Sparkle (Sp) and *brz* serving as negative and positive controls, respectively.

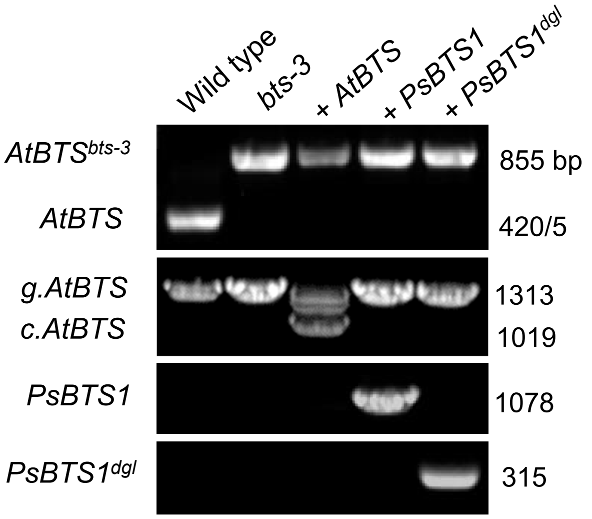

Supplemental Figure S6. Genotyping of the plants pictured in Figure 4b. The *bts-3* mutation removes a PflMI restriction site which can be detected by PCR followed by PflMI digestion. Sizes of the nucleotide bands are on the right. Primers are listed in Table S4.

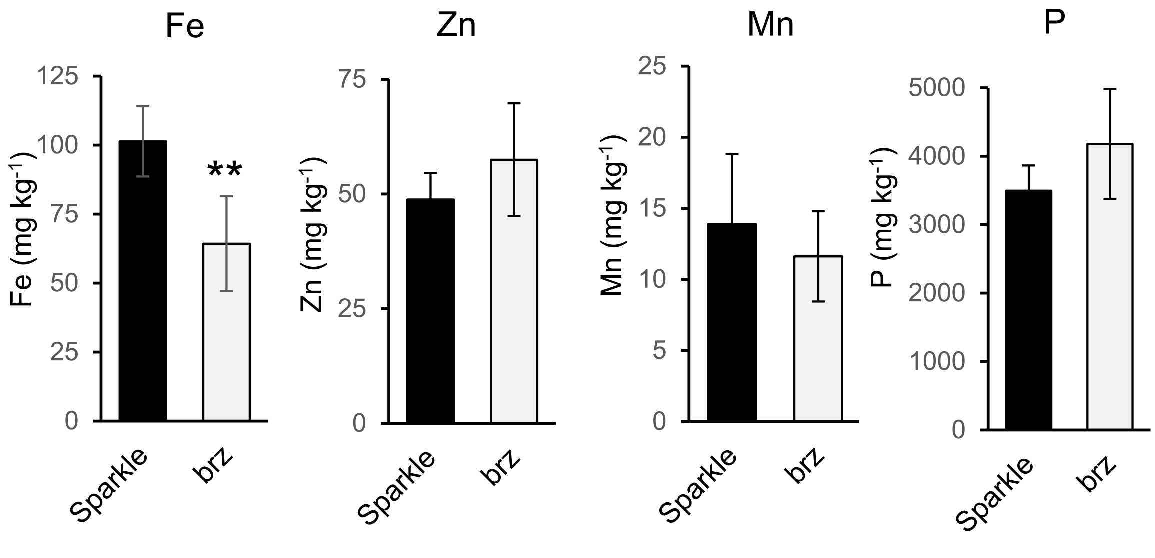

Supplemental Figure S7. Iron and zinc concentrations in pea seeds of *brz* compared to wild type. Seeds were collected from plants grown on compost. Iron (Fe), zinc (Zn), manganese (Mn) and phosphorus (P) were measured by Inductively Coupled Plasma-Optical Emission Spectroscopy. Values are the mean ± SD of representative seeds from 5 different plants. ** $P < 0.01$, Student t-test.

Supplemental Table S1. Gene Ontology terms of differentially expressed genes in leaves from the pea *dgl* mutant compared to the corresponding wild-type variety Sparkle. BP, biochemical pathway; MF, molecular function.

category	term	ontology	over_rep_padj
GO:0006826	iron ion transport	BP	3.58E-07
GO:0006879	cellular iron ion homeostasis	BP	4.95E-06
GO:0008199	ferric iron binding	MF	4.95E-06
GO:0055072	iron ion homeostasis	BP	4.95E-06
GO:0000041	transition metal ion transport	BP	1.28E-05
GO:0030003	cellular cation homeostasis	BP	2.25E-05
GO:0055082	cellular chemical homeostasis	BP	2.25E-05
GO:0006873	cellular ion homeostasis	BP	2.64E-05
GO:0050801	ion homeostasis	BP	8.73E-05
GO:0055080	cation homeostasis	BP	8.73E-05
GO:0048878	chemical homeostasis	BP	0.000207
GO:0030001	metal ion transport	BP	0.005288

Supplemental Table S2. Differentially expressed genes in leaves from the pea *dgl* mutant compared to the corresponding wild-type variety Sparkle.

Gene	q-value	Best Arabidopsis BLASTx hit	TPM in *dgl*	TPM in Sparkle	Change in TPM (*dgl* – Sparkle)
Psat1g183040	5.10E-29	GPT2 (AT1G61800)	7.20	64.88	-57.68
Psat1g112240	3.30E-23	AT5G51560	7.86	0.27	7.59
Psat6g186960	4.25E-23	None	60.30	0.00	60.30
Psat0s5295g0040	1.38E-15	ILL1 (AT5G56650)	53.91	0.00	53.91
Psat6g197040	9.85E-14	PTF2 (AT4G35540)	9.35	0.98	8.37
Psat2g029400	1.02E-12	None	34.64	0.00	34.64
Psat0s2407g0080	1.02E-12	None	34.64	0.00	34.64
Psat1g202520	1.70E-11	AT3G29750 and AT3G30770	0.08	1.08	-1.00
Psat6g193440	1.70E-11	None	37.44	0.00	37.44
Psat0s1206g0160	2.64E-09	AT1G48300	14.95	86.25	-71.30
Psat2g030160	4.52E-09	FERRITIN4 (AT2G40300)	529.82	156.43	373.39
Psat6g111680	4.52E-09	AT2G38970	1.60	0.00	1.59
Psat7g253960	2.41E-08	AT1G27670	12.26	0.35	11.90
Psat0ss2010g0120	2.74E-08	AT1G78230	0.01	2.49	-2.48
Psat1g060840	9.92E-08	DOX1 (AT3G01420)	1.20	71.04	-69.84
Psat1g097360	9.92E-08	Vacuolar iron transporter homolog 1 (AT1G21140)	20.35	4.66	15.69
Psat2g035000	1.14E-07	None	1.79	0.01	1.78
Psat1g166240	1.39E-07	PTF2 (AT4G35540)	20.33	5.70	14.64
Psat7g247120	4.61E-07	FERRITIN1 (AT5G01600)	3433.72	77.45	3356.26
Psat0s126g0080	3.81E-06	AT1G06720	0.00	6.46	-6.46
Psat4g186720	5.27E-06	Short hits to multiple ARF proteins	0.00	3.30	-3.30
Psat2g127760	1.48E-05	AT3G29180	0.00	0.59	-0.59
Psat7g253920	1.53E-05	None	24.54	3.66	20.88
Psat4g165600	1.71E-05	Short hits to multiple ARF proteins	17.53	0.00	17.53
Psat5g129280	1.71E-05	AT5G05600	1.11	6.54	-5.43
Psat5g295600	1.76E-05	IAA8 (AT2G22670)	14.55	32.96	-18.41
Psat6g190680	1.87E-05	RPS18C (AT4G09800)	99.72	33.70	66.02
Psat5g068840	3.24E-05	AT3G56360	9.02	105.01	-95.99
Psat6g194320	5.54E-05	AtRLP33 (AT3G05660)	0.00	1.13	-1.13
Psat6g188320	6.91E-05	None	67.24	18.13	49.10
Psat1g142960	8.77E-05	PIRL6 (AT2G19330)	10.61	3.14	7.46
Psat2g030280	0.00017	FERRITIN2 (AT3G11050)	352.03	107.15	244.87
Psat7g249400	0.00023	FBL3 (AT5G01720)	7.82	2.44	5.38
Psat1g162800	0.00023	None	0.01	1.94	-1.92
Psat5g159360	0.00057	AT1G67020	0.02	1.42	-1.40
Psat5g160280	0.00057	AT1G07280	27.33	51.63	-24.30
Psat5g187000	0.00057	None	9.55	0.34	9.21
Psat0s3850g0080	0.00057	GULLO6 (AT2G46760)	0.10	8.08	-7.98
Psat6g144720	0.00102	WDL1 (AT3G04630)	17.64	44.53	-26.89
Psat0s1271g0080	0.00129	AT5G23760	16.79	2.73	14.06
Psat2g146080	0.00193	ABCG14 (AT1G31770)	3.84	12.85	-9.00
Psat3g124720	0.00226	FERRITIN1 (AT5G01600)	147.83	5.13	142.70
Psat7g036120	0.00235	UGE1 (AT1G12780) and UGE3 (AT1G63180)	40.80	94.63	-53.83
Psat4g113920	0.00245	AT3G19430	3.06	39.81	-36.76
Psat5g214640	0.00250	AT4G14103	1.46	2.96	-1.50
Psat2g026880	0.00270	None	17.40	67.13	-49.73
Psat7g234040	0.00313	None	0.00	9.64	-9.64
Psat6g199320	0.00383	None	13.70	0.96	12.74
Psat7g067920	0.00385	FLA11 (AT5G03170)	2.24	22.82	-20.58
Psat0s4905g0040	0.00520	FLA12 (AT5G60490)	0.38	10.36	-9.98
Psat5g197640	0.00610	None	225.85	6.27	219.58
Psat7g009720	0.00682	SCD1 (AT1G49040)	3.70	9.03	-5.33
Psat7g120680	0.00689	None	68.35	29.95	38.40
Psat6g193360	0.00975	Vacuolar iron transporter homolog 2 (AT1G76800)	45.67	12.16	33.51
Psat1g098280	0.00998	CCD1 (AT3G63520)	16.98	0.00	16.98
Psat1g136120	0.00998	PFP-ALPHA1 (AT1G20950)	0.00	0.55	-0.55
Psat5g228400	0.01136	DER1 (AT4G29330)	46.87	11.92	34.95
Psat5g064800	0.01170	WRKY51 (AT5G64810)	7.38	2.12	5.26
Psat1g216120	0.01313	AMC1 (AT1G02170)	11.30	28.75	-17.45
Psat1g221440	0.01313	None	2.99	0.00	2.99

Supplemental Table S2, continued

Gene	q-value	Best Arabidopsis BLASTx hit	TPM in *dgl*	TPM in Sparkle	Change in TPM (*dgl* – Sparkle)
Psat7g099760	0.01759	EXL2 (AT5G64260)	0.22	1.67	-1.45
Psat7g099960	0.01778	MEE14 (AT2G15890)	5.49	23.92	-18.43
Psat4g008520	0.01859	None	302.35	41.61	260.74
Psat6g192200	0.01865	ARP1 (AT1G43170)	9.10	21.83	-12.73
Psat6g006240	0.02058	OBGL (AT5G18570)	10.23	6.31	3.92
Psat7g089640	0.02387	None	6528.98	22471.19	-15942.21
Psat7g091320	0.02387	ABCG11 (AT1G17840)	6528.98	22471.19	-15942.21
Psat0s3396g0040	0.02414	None	0.00	3.98	-3.98
Psat6g023960	0.02469	AT5G20670	16.37	39.92	-23.55
Psat5g098320	0.02566	ATMRP11 (AT2G07680)	0.60	1.59	-0.99
Psat7g009360	0.02702	PCR2 (AT1G14870)	44.77	6.74	38.04
Psat4g126840	0.02952	ILL4 (AT1G51760)	2.17	4.48	-2.31
Psat0ss8367g0160	0.02992	PSBA (ATCG00020)	55.98	122.47	-66.49
Psat3g171640	0.03079	AT3G01660	55.20	23.53	31.67
Psat0s9163g0080	0.03109	None	4.27	12.50	-8.22
Psat6g186240	0.03233	AT1G34340	3.64	0.99	2.65
Psat2g026840	0.03261	None	3.91	15.80	-11.89
Psat5g058520	0.03261	AT5G22450	0.73	0.15	0.58
Psat6g194200	0.03261	AT2G34930	0.02	0.10	-0.08
Psat5g213040	0.03325	AT3G51950	16.70	27.58	-10.89
Psat5g191320	0.03603	SERGT1 (AT3G01720)	0.00	0.63	-0.62
Psat1g111400	0.03683	YLS7 (TBL17, AT5G51640)	2.26	5.50	-3.25
Psat6g054320	0.03852	F9H_3 (AT4G03420)	0.40	1.73	-1.33
Psat1g005120	0.03886	None	12.50	0.00	12.50
Psat4g086600	0.04426	MAPKKK21 (AT4G36950)	1.52	7.08	-5.56
Psat5g124680	0.04654	PP2CA (AT3G11410)	3.35	7.86	-4.51

Supplemental Table S3 - TILLING mutations in *Medicago truncatula* genes.

Medicago OPT3 / Medtr4g133968	Amino acid change	Phenoptype in Medicago
c.G1219A	p.Asp407Asn	None
c.C1586T	p.Pro529Leu	Iron accumulation, bronze spots on older leaves, impaired growth
c.C2190T	p.Pro618Leu	Iron accumulation, bronze spots on older leaves, very impaired growth
c.C2265T	p.Ser643Phe	None
Medicago *BTS1* / *Medtr6g083900*		
c.G2188A	p.Asp730Asn	Embryo lethal
c.G2419A	p.Gly807Arg	None
c.G2734A	p.Asp912Asn	None
c.G2800A	p.Leu934Phe	None

Supplemental Table S4 - Primers used in this study.

Primer name	Sequence	Purpose
AtBTS-F	GCTATTCTAACCGTGAGA	Amplify a region encompassing the *bts-3* mutation, followed by PflMI digestion, which cleaves WT but not the *bts-3* allele.
AtBTS-R	AGAACCACAGCTACCAC	
JBSH167	GCGTGAAGAATGTAGCACAG	Detect the pea dgl allele based on PCR product length difference
JBSH168	ACCTGCAATATTCAACCAGCA	
M95	GTCCCTCCTTCTAACTCCG	Detect the *AtBTS* transgene in Arabidopsis
M103	TCCTTAGCCATGTGTTGACT	
M95	GTCCCTCCTTCTAACTCCG	Detect the *PsBTS1* transgene in Arabidopsis
M105	TTCATCAGTTTCCGTGGC	
M104	CCTTAGCCATGTGCTGC	Detect the *BTS1^{dgl}* transgene in Arabidopsis
JBSH190	ACGAGCTAGATGCGTTGCACCG	
MtOPT3-F1	CTTAGTCCTCTCTTCGCATTG	TILLING of Medicago *OPT3* target region
MtOPT3-R3	ACACCCATAAAAGCTGTG	
MtBTS1-F1	ATAGTCCGGTCTTCCTGTGC	TILLING of Medicago *BTS1* target region
MtBTS1-R3	GCCTTGCATTCACCACTATAG	
PsOPT3-F4	GACATATTGAGACGAGCAGG	Detect the pea *brz* mutation by PCR and Sanger sequencing
PsOPT3-R5	ATACCCAATCATGAACTGTGC	
